# Change in Hydrogen Trapping Characteristics and Influence on Hydrogen Embrittlement Sensitivity in a Medium-Carbon, High-Strength Steel: The Effects of Heat Treatments

**DOI:** 10.3390/ma17081854

**Published:** 2024-04-17

**Authors:** Zhi Tong, Hantong Wang, Wenyue Zheng, Hongyu Zhou

**Affiliations:** National Center for Materials Service Safety, University of Science and Technology Beijing, 30 Xueyuan Road, Haidian District, Beijing 100083, China; tongzhi@xs.ustb.edu.cn (Z.T.); wanghantong326@yahoo.com (H.W.)

**Keywords:** medium-carbon high-strength steel, heat treatment, hydrogen embrittlement, hydrogen trap

## Abstract

Medium-carbon, high-strength steels are widely used in the field of hydrogen energy because of their good mechanical properties, and they can be readily tailored by heat treatment processes such as the normalizing–tempering (N&T) and quenching–tempering (Q&T) methods. The hydrogen embrittlement (HE) susceptibility of a medium-carbon, high-strength steel was investigated utilizing microstructural characterization with scanning electron microscopy (SEM), the electron backscatter diffraction (EBSD) technique, and transmission electron microscopy (TEM). A study was also conducted on the steel’s hydrogen transport behavior as affected by the N&T and Q&T treatments. The steel contained more hydrogen traps, such as dislocations, grain boundaries, lath boundaries, and carbide interfaces, after the Q&T process, which was associated with a lower HE sensitivity when comparing the two treatments. In comparison, the N&T process produced larger-size and lesser-density carbides distributed along the grain boundaries, and this resulted in a relatively higher HE susceptibility, as revealed by the slow-strain-rate tensile (SSRT) tests of the hydrogen-charged steels and by the fractographic study of the fracture surface.

## 1. Introduction

Hydrogen is becoming the fastest-growing energy carrier in the world because it is renewable, widely available, and clean [1]. Due to its ability to achieve zero carbon dioxide emissions [2], hydrogen has been included in the national strategies for achieving future clean-energy development in countries all around the world [3,4]. Medium-carbon, high-strength steel can be used as the bolts, tanks, and valves used in hydrogen transportation and storage facilities. However, the strength and ductility of this class of steel are reduced due to the process of hydrogen degradation known as hydrogen embrittlement (HE) [5,6,7]. As the strength of the steel increases, the hydrogen embrittlement sensitivity becomes more pronounced [8,9,10,11,12]; therefore, the strength level of high-strength bolts has so far been limited to about >1000 MPa [13,14]. In a hydrogen-rich environment, once hydrogen enters the steel, it diffuses through the steel’s microstructure, but some of it is trapped in a “trap site”, which can reduce the overall rate of the hydrogen diffusion. Crack initiation and extensions occur when the material is subject to a sufficient enough stress in a hydrogen environment [15,16,17].

There are several proposed mechanisms for hydrogen embrittlement in steels, such as the hydrogen-enhanced decohesion (HEDE) mechanism, the hydrogen-enhanced localized plasticity (HELP), and adsorption-induced dislocation emission (AIDE). The HEDE mechanism mainly applies to cases where a material contains a high concentration of hydrogen and hydrogen atoms that are readily diffused within the material, thus reducing the interatomic or cohesive strength of the material at the crack tip, which leads to brittle fractures [18,19]. In the HELP model, the hydrogen gathering near the crack tip reduces the resistance to dislocation emissions and motion on the slip planes, as well as increases the dislocation mobility; in addition, the hydrogen-facilitated dislocation motion facilitates lattice plastic deformation [20,21]. Depending on the hydrogen concentration, the microstructure and the stress intensity at the crack tip may undergo various types of cracking such as intergranular, transgranular, and quasi-cleavage. Hydrogen promotes the emission of dislocations at the crack tip, which intensifies the crack extension by slip; as such, hydrogen can also influence the formation of microvoids through the HELP mechanism [21,22].

The difference in the microstructure of a steel directly determines its hydrogen embrittlement sensitivity. Adjusting the microstructure through a heat treatment process is a common way through which to improve the hydrogen embrittlement resistance of steel. N&T or Q&T are the two most common heat treatment processes, and they are widely used to control the microstructure of steel due to their ease of operation and low cost [23]. Imdad et al. [24] studied the HE sensitivity of 42CrMo4 steel after conducting annealed, normalized, quenched, and tempered heat treatments. Their research shows that the microstructures of bainite and pearlite are considered to be more susceptible to HE than the microstructures obtained via quenching and tempering. In addition, Zhou et al. [25] described the effects of N&T on the HE susceptibility of AISI 4300 steel, and they found that the interaction of hydrogen with the dislocations and nanoprecipitates in their steel produced evident HE effects. The difference in microstructure after N&T and Q&T treatment can result in a different susceptibility to hydrogen embrittlement for medium-carbon, high-strength steel, which will directly affect its service safety in a hydrogen-rich environment. Therefore, it is necessary to explore the influence of microstructure evolution and the hydrogen embrittlement sensitivity of medium-carbon, high-strength steel after N&T and Q&T treatment.

The aim of this work is to investigate, comparatively, the susceptibility of a medium-carbon steel to HE as it is affected by normalizing–tempering (N&T) and quenching–tempering (Q&T) treatments. This is achieved by studying the changes in its microstructure and the differences in hydrogen transport behavior. Exploring the mechanism of the hydrogen embrittlement of medium-carbon, high-strength steels after these two heat treatments is also important for material selection when determining appropriate applications for hydrogen-handling facilities.

## 2. Materials and Methods

The chemical composition of the medium-carbon, high-strength steel investigated in this work is listed in Table 1. It was alloyed with a small amount of Cr, Mo, and Ni, with a carbon content of 0.30%. Steel blocks (30 × 20 × 200 mm) were subjected to heat treatments that included austenitizing at 880/860 °C for 55 min, which were then subjected to air/water cooling at room temperature. Finally, the two samples were tempered at 600 °C for 67 min after normalizing and quenching. The samples that were obtained after the N&T and Q&T processes were named the NT and QT samples, respectively. In addition, the tensile strength of the NT and QT samples reached 1010.58 and 1124.37 MPa, respectively.

The samples for the microstructure characterization were prepared by wire-cut electrical discharge machining. These samples were ground by 400- to 2000-grit SiC papers under running water and polished by a diamond polishing paste of W2.5. Subsequently all the samples were etched with a 4% vol. nital solution. The microstructural examinations of the samples were conducted using scanning electron microscopy (SEM, Zeiss Merlin Compact, Germany).

The electron backscatter diffraction (EBSD, Nordlys Max3, UK) samples were subjected to standard mechanical polishing, which was followed by electropolishing in a solution of 10% vol. perchloric acid, 85% vol. acetic acid, and 5% vol. glycerin. Perchloric acid can form high-viscosity and high-resistance complexes with the many metals and other negative ions in the solution used in this study, thus resulting in polarization. Perchloric acid also has a good polishing effect on many metals, so it is widely used to prepare EBSD samples. The details of the working conditions for EBSD analysis were as follows: the accelerating voltage was 20 kV, the tilt angle was 70°, and the step size was 0.5 μm.

The carbon extraction replica technique was applied to characterize the precipitates in the steel. The specimens were firstly polished etched with 2% vol. nital; then, they were covered by evaporated carbon, extracted, and finally placed on the copper grid to be analyzed. These carbon replicas were analyzed by transmission electron microscopy (TEM, JEM2011f, UK) at 200 kV with energy dispersive spectroscopy (EDX) mapping. TEM was conducted using a JEM-2200FS (UK) at 200 kV. In addition, TEM was also performed on the foils, which were prepared by cutting thin discs from 3 mm-thick cylinders that were machined in the center of their walls. The small discs were ground to a ~50 μm thickness and then electropolished using an electrolyte containing 10% perchloric acid in acetic acid.

A Devanathan–Stachurski cell with anodic and cathodic compartments was used in hydrogen permeation conditions through the membranes of the test steel [26]. The specimen was ~1.28 mm in thickness, and the area of the hydrogen permeation was 1.77 cm^2^. On the side of the anode chamber, a calomel electrode was used as the reference electrode, and the sample was used as the working electrode. The anode chamber was filled with 0.2 mol/L of a NaOH solution with an applied potential of 200 mV. The solution (0.2 mol/L of NaOH + 0.25 g/L of thiourea) was added into the cathode chamber, and hydrogen charging was carried out with a current density of 2 mA/cm^2^. Platinum sheets were used as the counter electrodes in both cells.

The hydrogen trapping sites were analyzed by thermal desorption spectroscopy (TDS) [27]. The samples for TDS (JTF20A Series, Japan), which had dimensions of 10 mm × 10 mm × 5 mm, were polished to a 5000-grit level using SiC papers, and they were then cleaned ultrasonically in ethanol. Subsequently, the specimens were electrochemically charged with hydrogen in 0.2 mol/L of NaOH + 0.25 g/L of thiourea for 72 h at a current density of 2 mA/cm^2^ to ensure hydrogen saturation in the steel. The TDS experiments were performed with quadrupole mass spectroscopy at a heating rate of 200 °C/h, and the vacuum level was ~10^−11^ Pa.

**Figure 1:**
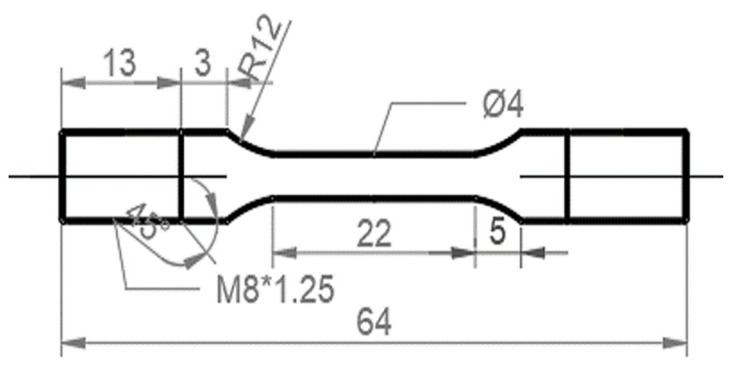
Geometry of the SSRT test specimens (unit: mm).

Slow-strain-rate tensile (SSRT) tests were conducted to evaluate the HE susceptibility of the H-charged specimens. The specimens for the SSRT tests were cut by electro discharge machining, the geometry of which is shown in Figure 1. The samples were ground to a 2000-grit level using SiC papers and then cleaned ultrasonically in ethanol. SSRT tests of the uncharged and pre-charged hydrogen samples were performed on a WEML-25 tensile machine at a strain rate of 10^−5^/s with a preload of 400 N. The electrochemical hydrogen-charging solution used was a deionized aqueous solution of 0.2 mol/L of NaOH + 0.25 g/L of thiourea. Before the SSRT tests started, the sample was pre-charged with hydrogen, and the current density of the pre-charging was 2 mA/cm^2^ for 24 h. The SSRT tests were commenced immediately after hydrogen charging.

## 3. Results

### 3.1. Hydrogen Embrittlement Susceptibility

**Figure 2:**
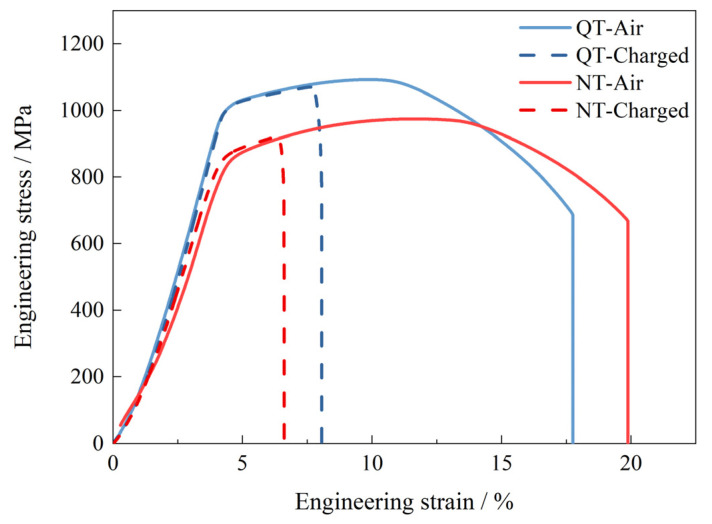
The curves for the SSRT tests of the hydrogen-uncharged (air) and hydrogen-charged samples at 2 mA/cm^2^ for 24 h.

The HE sensitivity of the SSRT test samples was used to evaluate the influence of hydrogen on the mechanical property after the N&T and Q&T treatments. The hydrogen sensitivity, *I_HE_*, is expressed by the following [28]:

(1) $$I_{HE}(\%)=\frac{\delta_{0}-\delta_{H}}{\delta_{0}}\times 100\%,$$

where *δ*_0_ and *δ_H_* are the elongations of the SSRT tests without and with hydrogen charging, respectively. Figure 2 shows the tensile test curves for the NT and QT samples with and without hydrogen charging (2 mA/cm^2^ for 24 h). During hydrogen charging, the yield strength of the specimens remained relatively invariant; however, a pronounced deterioration in the elongation performance was observed as a result of hydrogen exposure. The QT specimen demonstrated a reduction in its elongation from 17.7% in ambient air to 8.1% under hydrogen-charging conditions. Similarly, the NT specimen exhibited a decrease in its elongation from 19.9% in air to 6.6% under hydrogen charging. Compared with the test in air, the yield strength of the NT and QT samples did not change significantly under hydrogen-charging conditions; however, the elongation was greatly reduced, and this reflected the effects of the hydrogen that had entered the steel microstructures. Based on Equation (1) and the engineering stress–strain curves, the HE susceptibility of the NT and QT samples were calculated to be 66.80% and 54.64%, respectively. The hydrogen-charged QT sample had a better ductility than the NT steel, and it also showed a lower hydrogen embrittlement sensitivity.

### 3.2. Microstructure Characterization

**Figure 3:**
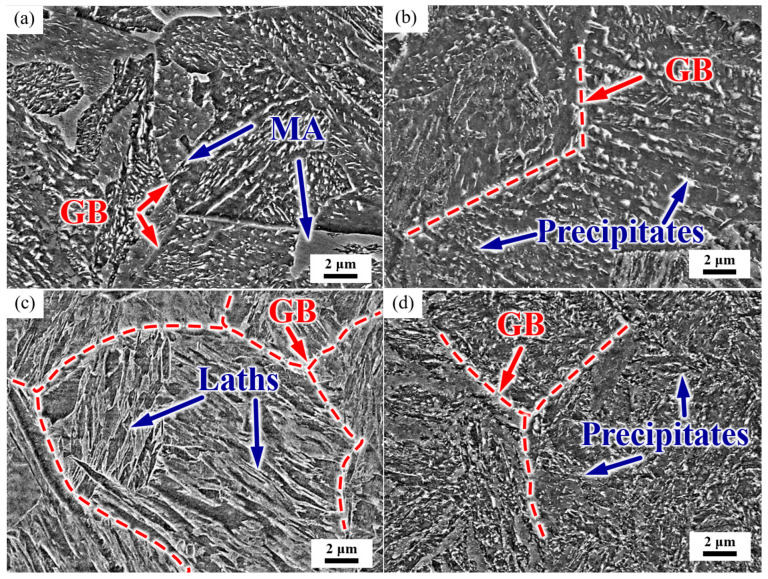
Representative SEM micrograph of the microstructure. (**a**) As-normalized, (**b**) NT, (**c**) as-quenched, and (**d**) QT. GB: grain boundary, MA: martensite–austenite constituent. Red dashed lines represent the PAGBs.

The microstructure of the test steel is shown in Figure 3. After normalizing, the microstructure was mainly of a granular bainite with clear grain boundaries. The intracrystalline martensite–austenite (MA) constituent was discontinuously distributed in some grains, and a small amount of large blocky MA was present at the grain boundaries (Figure 3a). After the subsequent tempering process, the prior austenite grain boundaries (PAGB) were gradually blurred, the MA decomposed, and a large number of precipitates appeared in the PAGB and the interior of the grain (Figure 3b). The steel was then quenched, and the microstructure was lath martensite, with grain boundaries and martensite laths clearly visible (Figure 3c). After tempering, the grain boundaries were gradually blurred, and the precipitates were distributed along the martensite laths and PAGBs (Figure 3d).

**Figure 4:**
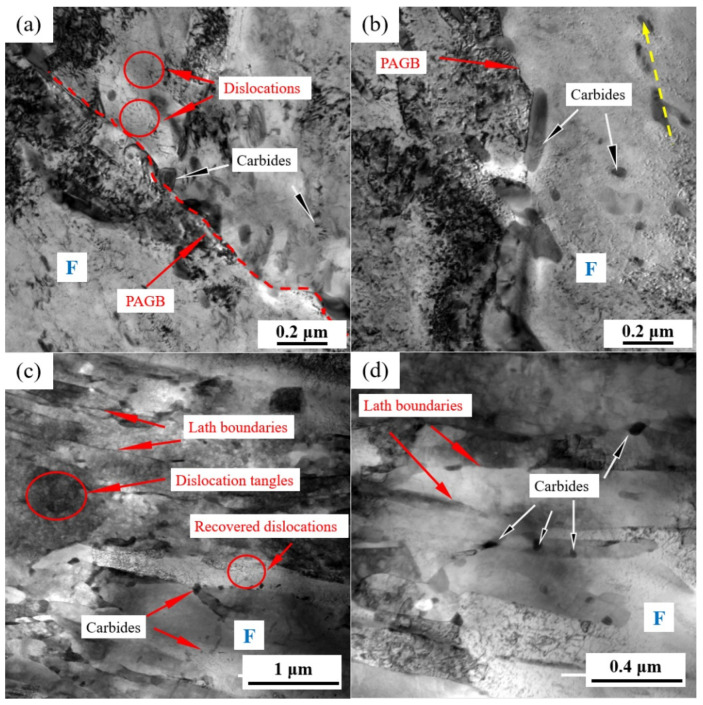
TEM micrographs showing the (**a**,**c**) morphology and dislocations, and (**b**,**d**) carbides in the NT (**a**,**b**) and QT (**c**,**d**) samples. The yellow arrow represents the orientation of the precipitate. The red dashed line represents the PAGB. PAGB: prior austenite grain boundary and F: ferrite.

Figure 4 shows the typical grain morphology, dislocation, and carbides in the NT and QT samples that were, respectively, observed by TEM. The microstructure of the NT sample consisted of a predominant ferrite matrix and some carbides, with a few dislocation lines distributed in the ferrite matrix (Figure 4a). Carbides were mainly located in the PAGBs and the interior of the grains. Examples of the carbides in the NT sample are shown in Figure 4b, and they were mainly divided into the following two categories: (1) the block or rod-shaped carbides distributed in the PAGBs or prior to MA (Figure 4b) with an average long axe of 140~280 nm and short axe of 40~100 nm, and (2) a small amount of fine round carbides, with an average diameter ≤60 nm, which were distributed in the ferrite matrix.

A typical TEM morphology with the dislocations and precipitates of the QT sample is shown in Figure 4c,d. The prior martensite laths can still be clearly observed with a lath width of approximately 110~340 nm, and these formed a fully tempered martensite structure, as shown in Figure 4c. A large number of dislocation tangles and some recovered dislocations can be observed in the ferrite matrix, which indicated that a higher dislocation density was present in the QT sample (the quantitative data of which are shown in the next section). The carbides are shown in Figure 4d. The carbides were mainly discontinuously distributed at the interface of the prior martensite lath with sizes in the range of 28.28~59.21 nm, and their morphology was blocky. Additionally, a small number of carbides were also observed within the subgrains.

**Figure 5:**
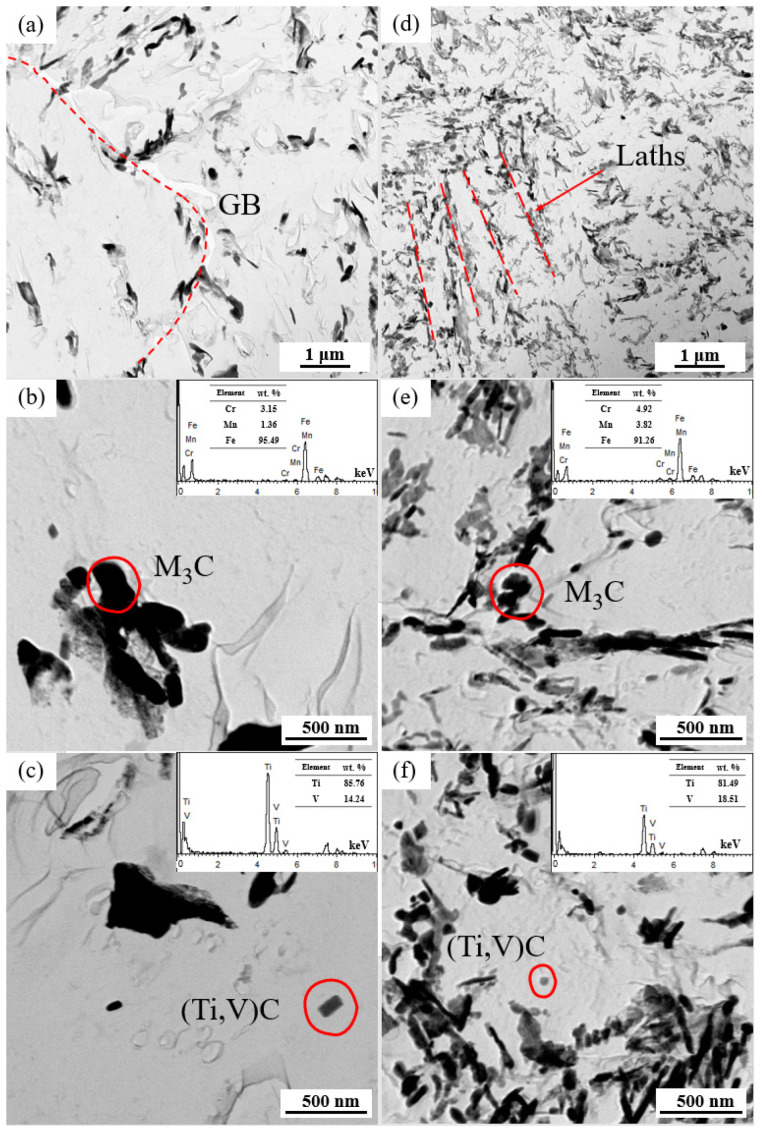
The TEM bright fields in the pictures in the replicas of the NT (**a**–**c**) and QT (**d**–**f**) samples. The carbides with respective EDXs for the NT (**b**,**c**) and QT (**e**,**f**) samples. The red dashed line represents the orientation of the carbides.GB: grain boundary.

The distributions of the carbides in the replica samples examined by TEM are presented in Figure 5. Compared to the NT samples, the QT samples exhibited a more homogenous distribution (Figure 5a,d). The NT samples mainly had carbides in the form of large blocks at the grain boundaries, while the carbides in the QT samples were mainly distributed at the interfaces of the prior martensite lath. These larger carbides were identified as cementite M3C (M = Fe, Mn, and Cr) by EDX analysis and physicochemical phase analysis, as verified elsewhere (Figure 5b,e) [29]. Spherical or elongated V/Ti-rich carbides (Ti,V)C were sporadically contained in the ferrite matrix (Figure 5c,f). These carbides were mainly nanoscale particles, which may have occurred due to the undissolved carbides that were prevented from austenitizing and because these (Ti,V)C dissolution temperatures were higher than the austenitizing temperatures (880/860 °C) applied in this work.

**Figure 6:**
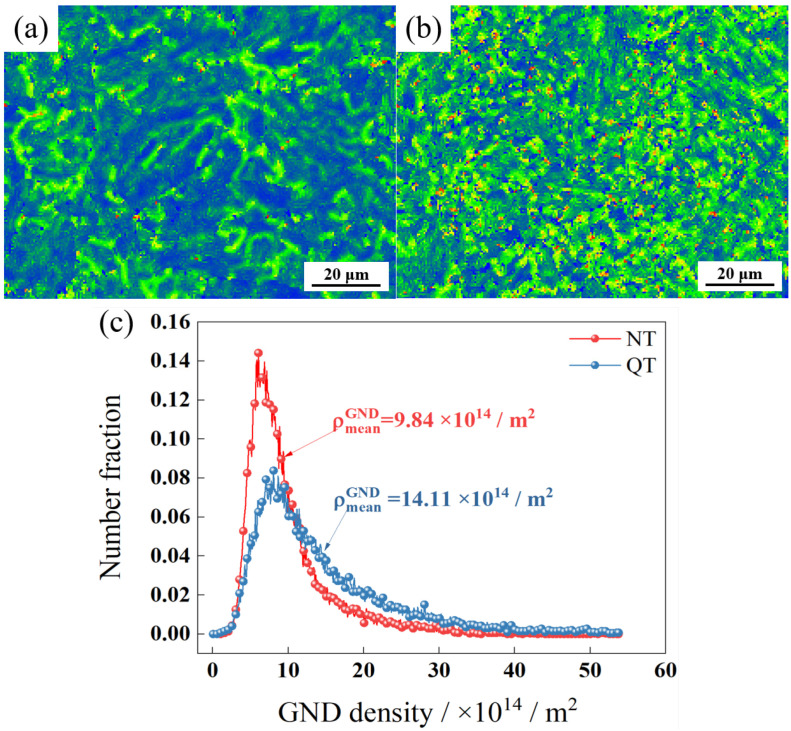
The KAM of (**a**) NT and (**b**) QT samples and (**c**) GND density.

Figure 6a,b, respectively, show the internal strain (kernel average misorientation, KAM) of the NT and QT samples. The KAM value is the average misorientation angle between a given point and its neighbors [30]. Figure 6c shows the geometrically necessary dislocation (GND) density that was calculated by EBSD analysis. ρ_mean_^GND^ represents the average geometrically necessary dislocation density of the whole region. The NT sample had a higher percentage of smaller GND density regions, while the QT sample had a higher average GND density with a value of ~14.11 × 10^14^/m^2^.

**Figure 7:**
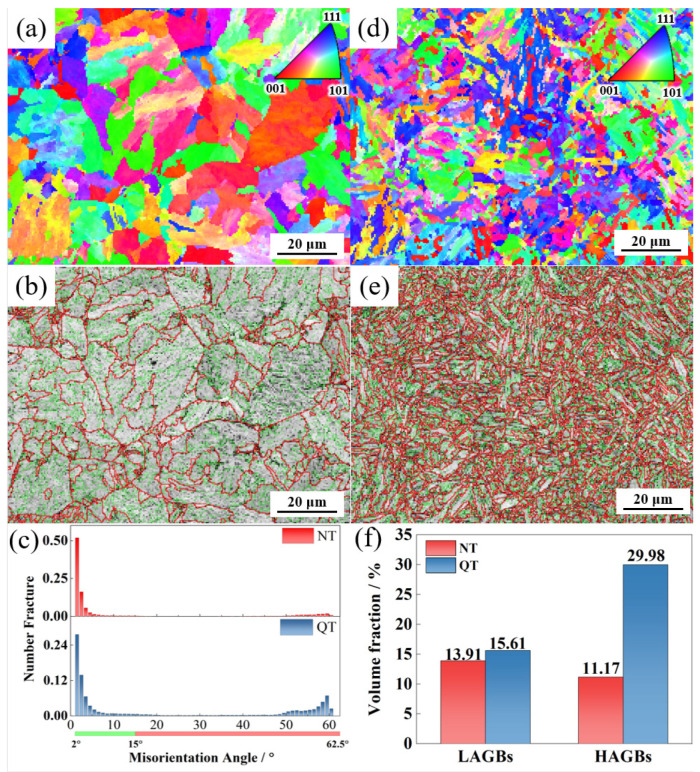
The IPF and grain boundary maps in the (**a**,**b**) NT and (**d**,**e**) QT samples. (**c**,**f**) The distribution and volume fraction of each boundary in the NT and QT samples. LAGBs: 2°~15° (green line) and HAGBs: ≥15° (red line).

Figure 7 shows the crystal orientation (inverse pole figure, IPF) and grain misorientation angle distribution of the NT and QT samples. Each color in the IPF diagram was determined according to the deviation from the measured orientation. Grain boundaries with orientation angles of 2° < θ < 15° and 15° < θ are defined as low-angle grain boundaries (LAGB) and high-angle grain boundaries (HAGB), respectively, and they are represented by green and red solid lines. Figure 7a,d show that the QT samples had a more homogenous and finer microstructure. From the comparisons shown in Figure 7b,e, it can be seen that the QT sample had a larger density of HAGBs compared with the NT sample (Figure 7c). A comparison of the volume fraction of the grains in the two steels with LAGBs and the HAGBs indicated that the QT sample contained 29.98% grains with HAGBs in its microstructure while the NT sample had 11.17%; in addition, the LAGBs were also higher in the QT sample than in the NT sample (15.61% versus 13.91%).

### 3.3. Hydrogen Permeation and Hydrogen Trapping Sites

**Figure 8:**
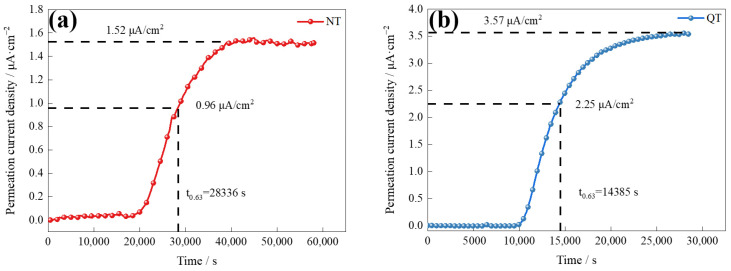
The electrochemical hydrogen permeation curves of the (**a**) NT and (**b**) QT samples.

Figure 8 shows the curves of the electrochemical hydrogen permeation of the NT and QT samples at a 2 mA/cm^2^ charge current density. The hydrogen flux rate is defined by [31]

(2) $$J_{H}L=I_{\infty }L/FA,$$

where *J_H_L* is hydrogen permeability, *I*_∞_/*A* is the saturation current density measured in the hydrogen flux curve, *L* is the specimen thickness, and *F* is the Faraday constant (96,500 C/mol). The effective hydrogen diffusivity, *D_eff_*, can be calculated by [32,33]

(3) $$D_{eff}=L^{2}/6t_{L},$$

where *t_L_* is the time for the hydrogen flux to reach 63% of the final saturation (steady state) level. The apparent hydrogen solubility at the hydrogen entrance side, *C_app_*, is then defined by

(4) $$C_{app}=J_{H}L/D_{eff}.\;$$

The results of the hydrogen permeation curves reveal the details of the hydrogen diffusion and hydrogen trapping features of the two samples, and the parameters and results of the hydrogen permeation are shown in Table 2. The QT sample had a higher hydrogen diffusion coefficient (1.880 × 10^−7^ cm^2^/s), which was about twice that of the NT sample. The surface hydrogen concentration (*C_app_*) at the hydrogen-charging side of the QT sample was 0.42 × 10^−5^ mol/cm^3^ (or about 20%) higher than that of the NT sample. The diffusion and solubility of the hydrogen inside the NT and QT samples were affected by grain boundaries, dislocations, martensite lath boundaries, and carbide interfaces [34].

**Figure 9:**
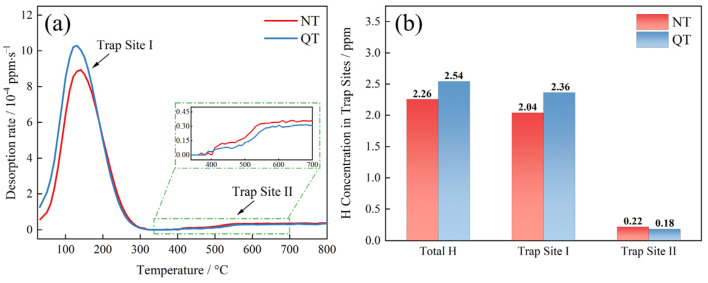
(**a**) TDS spectra under a 200 °C/h heating rate and (**b**) the accumulation of hydrogen concentration in the specimens.

The TDS spectrum and accumulated hydrogen at a heating rate of 200 °C/h are shown in Figure 9. A clear low temperature peak (Trap Site I), which corresponded to a temperature of about 135 °C, was present in both samples (Figure 9a). The QT sample had an overall higher desorption rate (reaching 10.29 × 10^−4^ ppm/s) than the NT sample (8.94 × 10^−4^ ppm/s). The trapping sites were the reversible hydrogen trap type at this temperature, and its activation energy was about 15–35 kJ/mol [35]. A high temperature site (Trap Site II) was found at a temperature of 350 °C with a high binding force with hydrogen, which is considered to be an irreversible type of hydrogen trap. 

The sites of the total hydrogen concentration trapped by each type of hydrogen trap are shown in Figure 9b. The total hydrogen concentrations in the NT and QT samples were 2.26 ppm and 2.54 ppm, respectively. The TDS data show that the hydrogen atoms were mainly trapped at Trap Site I with concentrations of 2.04 ppm and 2.36 ppm, respectively. A small amount of hydrogen atoms was trapped by Trap Site II, with concentration of 0.22 ppm and 0.18 ppm, respectively.

**Figure 10:**
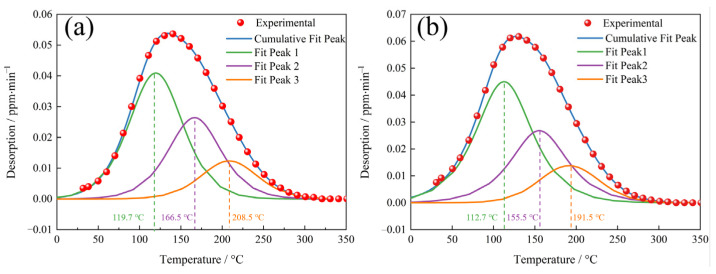
The deconvolution of the low temperature (≤350 °C) TDS peak for the (**a**) NT and (**b**) QT samples.

To determine the hydrogen trapping characteristics of each hydrogen trap, the low temperature peak (≤350 °C) spectra of the TDS, as shown in Figure 9, were deconvoluted into three Gaussian curves in accordance with Gong [36], and the deconvolution results are shown in Figure 10. The hydrogen concentrations of each fit peak and the total hydrogen concentrations were calculated based on the deconvolution curves, and the results are shown in Table 3. The QT samples had, as expected, higher hydrogen concentrations at each fitted peak compared to the NT samples.

### 3.4. HE Fracture Surface Characteristics

**Figure 11:**
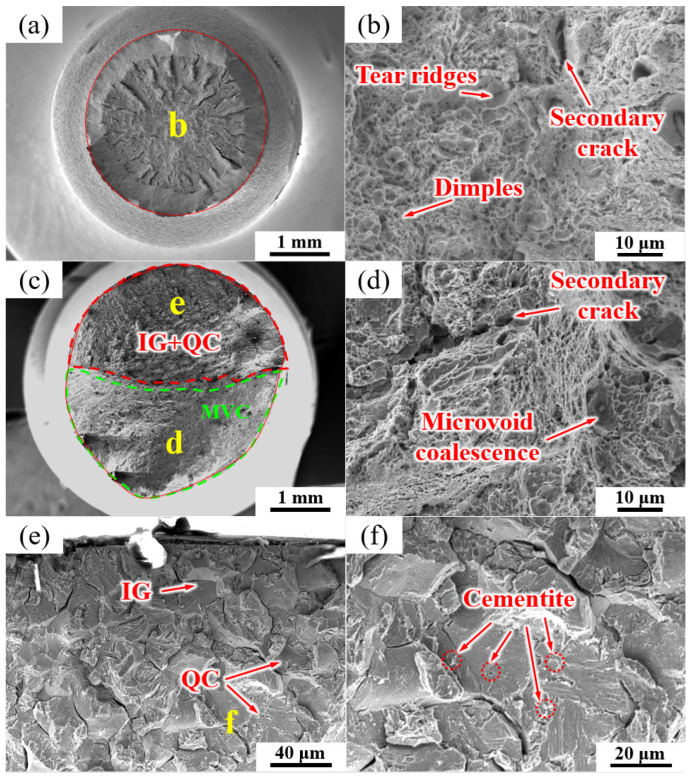
SEM micrographs of the fracture surfaces of the NT sample (**a**, **b**) without and (**c**–**f**) with hydrogen charging. The labels (**d**–**f**) show the high magnification images of regions (**d**–**f**) in images (**c**,**e**), respectively.

The fracture morphologies of the NT sample under air and hydrogen-charging conditions are shown in Figure 11. In the absence of hydrogen charging, the fracture morphology exhibited a typical ductile overload fracture mode, and the fracture morphology was mainly composed of dimples (Figure 11a,b) with a few secondary crack-like features. Figure 11c–f show the fracture mode of the NT fracture specimen after hydrogen charging. In Figure 11c, a mixture of intergranular cleavage (IG), quasi-cleavage (QC) and microvoid coalescence (MVC) fracture modes were observed. IG + QC and MVC modes each occupied approximately half of the fracture area.

Figure 11d shows an enlarged view of the green area in Figure 11c, which was mainly composed of the MVC mode and a few secondary cracks. Figure 11e shows an enlarged image of the region enclosed by the red line in Figure 11c, as well as the intergranular fractures along the PAGB, QC, and partial secondary cracking. Some cementite particles can be seen on the intergranular fractures of the PAGB and QC facets (Figure 11f).

**Figure 12:**
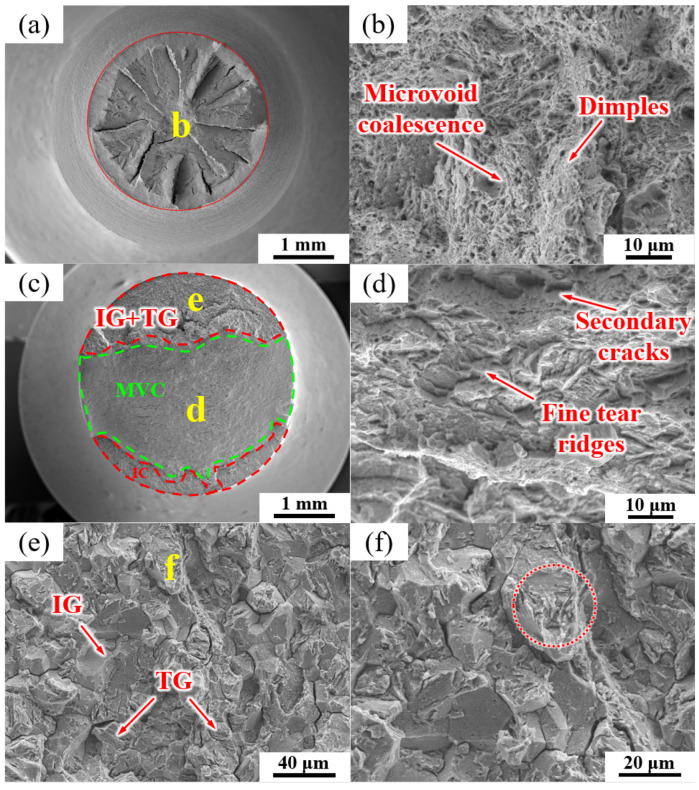
SEM micrographs of the fracture surfaces of the QT sample (**a**,**b**) without and (**c**–**f**) with hydrogen charging. (**d**–**f**) are the high-magnification images of regions (**d**–**f**) in images (**c**,**e**), respectively.

The fracture morphologies of the QT sample under air and hydrogen-charging conditions are shown in Figure 12. The fracture morphology without hydrogen charging was mainly composed of dimples and MVC (Figure 12a,b). Figure 12c–f show the fracture surface of the SSRT fractures after hydrogen charging the QT sample. A mixture of IG, TG (transgranular cleavage), and MVC fracture modes was observed, as shown in Figure 12c, and about 1/3 of the fracture surface consisted of the IG + TG facets. Figure 12d is an enlarged image of the green area in Figure 12c, which is mainly composed of the MVC mode with a small number of secondary cracks and fine tear ridges. 

Figure 12e is an enlarged view of the region defined by the red line in Figure 12c, which shows a mixture of TG (alongside martensite lath) fractures and intergranular fractures along the PAGBs. It can be clearly observed that a few TG cracks also propagated along the prior martensite lath (see red circle in Figure 12f).

## 4. Discussion

### 4.1. Hydrogen Trapping and Diffusion

The TDS spectra in Figure 8, Figure 9 and Figure 10 describe the hydrogen trapping features for each type of hydrogen trap in the two steels. The low temperature peaks represent the trap sites that had lower binding energies with hydrogen. The high temperature peaks (>350 °C) were irreversible hydrogen traps, which are generally considered to be carbides of Fe and alloying elements (including Nb, Ti, and V) with activation energies that are generally above 50 kJ/mol [37,38,39]. The results of our TDS measurements showed that the hydrogen was mostly trapped by the reversible hydrogen traps in the test steel in the NT and QT samples (Figure 9a).

The low temperature peak was deconvoluted into three peaks (Figure 10), where the first peak corresponded to the lowest activation energy. Fit Peak #1 was considered to be associated with the dislocations and LAGBs, and the corresponding activation energy was 20 ≤ Eb ≤ 35 kJ/mol [40,41,42,43]. The hydrogen concentrations captured by Peak #1 in the QT and NT samples were 1.05 wppm and 1.22 wppm, respectively. After quenching, the sample had lath martensite with a high density of dislocations. The dislocation density of the QT sample was still higher at ~14.11 × 10^14^/m^2^, even though some of the dislocations were annihilated at a tempering of 600 °C. The QT sample had a higher dislocation density than the NT sample. In addition, the two steels had similar volume fractions of LAGBs (~13.91% in the NT sample and ~15.61% in the QT sample), and this explains why more hydrogen was trapped by Peak #1 in the QT sample.

The second deconvolution peak was considered to be related to boundaries [40,41,42,43] such as grain boundaries and martensite boundaries. In the austenitizing process, in which the two types of samples had similar heating temperature and the same holding time, it can be assumed that the prior austenite grains were of similar size in both the NT and QT samples. Thus, the NT and QT samples should have the same volume fraction of PAGBs. After the tempering process, the prior martensitic lath interface was still clearly visible (Figure 4c). However, in the TDS, one could not definitively distinguish between the PAGB and the prior martensitic lath trapping sites [44,45], so the volume fraction of the HAGBs in the QT sample (~29.89%) was about three times that of the NT sample (~11.17%). Thus, the HAGBs in the QT samples could be counted on to capture more H atoms. And the fine carbides were evenly distributed at the HAGBs, which can cause reductions in the overall available H that can be captured at HAGBs.

The third peak of the low temperature peak after deconvolution should be associated with coherent precipitates. In this study, the cementites can be considered as coherent precipitates. These cementites were mainly distributed at the grain boundary, the prior MA interface, and the prior martensite lath interface (Figure 4 and Figure 5). Cementite represented the hydrogen trap with the highest activation energy in the group of reversible H traps (Figure 10). As hydrogen enters a sample, it will preferentially diffuse into the cementite sites. In comparison, carbides such as (Ti,V)C with less volume fraction in the NT and QT samples (Figure 5c,f) were considered irreversible hydrogen traps, which were indicated as a high temperature peak in the TDS spectrum. However, these irreversible hydrogen traps with strong trap binding energies were generally considered resistant to causing HE [46].

Generally, the diffusion coefficient of hydrogen can be reduced by more reversible hydrogen traps [47]. For example, cementite, which is an effective H trap, can effectively reduce the diffusion coefficient of H, as was reported by Pinson in their study of martensitic medium-carbon steels [48]. In addition, the smaller the size of carbides, the smaller the average free path for H, which means that the overall diffusion path for H atoms to transverse steel structures is increased, thus lowering the apparent overall diffusion coefficient [49]. Compared with the NT sample, the QT sample not only had smaller carbides (Figure 5), but it also had more dislocations (Figure 6). These reversible hydrogen traps should, in theory, hinder the diffusion of hydrogen. However, according to the hydrogen permeation tests, the QT sample had a surprisingly higher diffusion coefficient, where the hydrogen diffusion coefficients of the QT and NT samples were 1.880 × 10^−7^ cm^2^/s and 0.968 × 10^−7^ cm^2^/s, respectively. This was mainly due to the fact that the diffusion of hydrogen in the steel was likely controlled by the competition between the short-circuit diffusion along random grain boundaries and the hydrogen trapping at the dislocations [50]. Since the tempered QT sample still, even after tempering, retained the lath morphology of the martensite that was formed by quenching—and as these lath interfaces become excellent short-circuit diffusion paths for hydrogen—it evidently resulted in a higher hydrogen diffusion coefficient in the QT sample in comparison with the NT sample.

### 4.2. Hydrogen Embrittlement Mechanism

The hydrogen-related parameters of NT and QT samples are summarized in Table 4. The SSRT tests revealed a difference in the hydrogen embrittlement sensitivity of the NT and QT samples. The results showed that the QT sample had a lower hydrogen embrittlement sensitivity of ~54.64 % (Table 4). In this work, due to the small number of (Ti,V)C in the steel, only a small amount of hydrogen was captured by such carbides [46]. Therefore, the effect of (Ti,V)C on the HE sensitivity in the steel can be ignored. The deepest hydrogen traps among the reversible traps in this work were cementites (Figure 10). When enough hydrogen diffuses to cementite sites, hydrogen may cause the cementite interface to spall off from the matrix under the action of stress [48]. In general, a crack usually propagates along the path with the most minimum resistance toward fractures [51]. Cementites with weakened interfaces become the preferred sites for the initiation of cracks. The path with the weakest fracture propagation resistance in the NT sample was PAGB. In addition, with the entry of hydrogen, the cementites at such a type of grain boundary promoted a de-bonding at the interfaces of the PAGB, thus resulting in IG fractures (Figure 11e,f), and this was consistent with the HEDE mechanism. The quasi-cleavage (QC) fracture mode was also observed in the fracture surfaces of the NT sample (Figure 11e,f). For the QT sample, the weakest crack propagation resistance was the HAGBs (i.e., the PAGB and the martensite lath interface). The initiation and propagation of the cracks were enhanced at such interfaces by the HEDE mechanism, which led to IG fractures along the PAGB (Figure 12e,f) [52,53,54].

HEDE is the main mode of failure in high-strength steel [55]. The HEDE mechanism can be activated only when the local hydrogen concentration at the interface reaches a critical value [56,57]. Although the total hydrogen concentration of the QT sample in the interface was higher than that of NT sample (Table 3), the QT sample had a much higher number of martensite lath interfaces, which were regarded as HAGBs (Figure 7). This large number of martensite lathes increased the effective total net area of the grain boundary, and so it effectively reduced the actual concentration of hydrogen per unit area of the lath interface; therefore, the HE cracking sensitivity was mitigated following the HEDE mechanism [58]. This explains why the QT sample had less of an IG fracture facet area (Figure 11c and Figure 12c) with lower HE sensitivity. Moreover, a larger number of dispersed fine cementite (Figure 4 and Figure 5) in the QT sample increased the hydrogen trap density in the steel, thus resulting in a more uniform hydrogen distribution, which is also beneficial for reductions in the overall HE sensitivity.

## 5. Conclusions

The medium-carbon, high-strength steel was obtained by N&T and Q&T heat treatment processes. The effects of the two heat treatment processes on the samples’ hydrogen embrittlement sensitivity and the trapping characteristics were comparatively investigated, and the conclusions are as follows:

(1) After the N&T and Q&T heat treatments, both the NT and QT samples were found to have dislocations, grain boundaries, and carbides as hydrogen traps. The dislocation density was 9.84 and 14.11 × 10^14^/m^2^, and the volume fraction of the HAGBs was 11.7% and 29.98%, respectively. The carbides in the NT sample were distributed at the PAGBs and prior MA interface with a larger size, and the fine carbides in the QT sample were distributed dispersedly at the interface of the PAGBs and prior martensite lath interface.
(2) Through TDS and deconvolution analysis, the hydrogen concentrations trapped by dislocations, interfaces, and carbides in the NT and QT samples were 1.05/1.22, 0.68/0.76, and 0.33/0.38 wppm, respectively. And the hydrogen diffusion coefficients in the NT and QT samples were confirmed to be 0.968 × 10^−7^ and 1.880 × 10^−7^ cm^2^/s, respectively.
(3) The HE susceptibility of the NT and QT samples were 66.80% and 54.64%, respectively. Compared with the NT sample, the QT sampled had a lower hydrogen embrittlement sensitivity. The dispersed and fine carbide distribution, as well as the greater number of martensite lath interfaces in the QT sample, contributed to a relatively lower susceptibility to hydrogen embrittlement.

## Figures and Tables

**Table 1:** Chemical composition of the experimental steel (wt. %).

C	Si	Mn	Cr	Ni	Mo	V	Ti	Cu	P	S	Fe
0.30	0.25	0.85	0.90	1.65	0.25	0.08	0.005	0.188	0.0088	0.005	Bal.

**Table 2:** The hydrogen permeation parameters of the NT and QT samples.

No.	L/cm	t_0.63_/s	*D_eff_*/10^−7^ cm^2^·s^−1^	*C_app_*/10^−5^ mol·cm^3^
NT	0.1283	28,336	0.968	2.09
QT	0.1275	14,385	1.880	2.51

**Table 3:** The hydrogen concentration of the deconvoluted low temperature peaks shown in Figure 9 (wppm).

No.	Fit Peak 1	Fit Peak 2	Fit Peak 3	Total H—Fitted	Total H—Measured
NT	1.05	0.68	0.33	2.06	2.04
QT	1.22	0.76	0.38	2.36	2.36

**Table 4:** Summary of the hydrogen-related parameters of the NT and QT samples.

No.	ρ_mean_^GND^/m^−2^	HAGBs/%	*D_app_*/×10^−7^ cm^2^·s^−1^	*I_HE_*/%
NT	9.84	11.70	0.968	66.80%
QT	14.11	29.98	1.880	54.64%

## Data Availability

Data are contained within the article.
